# Potent anticancer activity of (Z)-3-hexenyl-*β*-_D_-glucopyranoside in pancreatic cancer cells

**DOI:** 10.1007/s00210-023-02755-4

**Published:** 2023-10-11

**Authors:** Ahmed M. Zaher, Walaa S. Anwar, Makboul A. Makboul, Iman A. M. Abdel-Rahman

**Affiliations:** 1 Department of Pharmacognosy, Faculty of Pharmacy, Assiut University, Assiut, 71515 Egypt; 2 Department of Pharmacognosy, Faculty of Pharmacy, Merit University, New Sohag, Egypt; 3 Department of Pharmacognosy, Faculty of Pharmacy, South Valley University, Qena, Egypt

**Keywords:** (Z)-3-hexenyl-*β*-D-glucopyranoside, Casp3, Bax, Bcl-2, Panc1

## Abstract

This current study reports, for the first time, on the potent cytotoxicity of (Z)-3-hexenyl-*β*-_D_-glucopyranoside, as well as its cellular and molecular apoptotic mechanisms against Panc1 cancer cells. The cytotoxicity of three compounds, namely (Z)-3-hexenyl-*β*-_D_-glucopyranoside (1), gallic acid (2), and pyrogallol (3), which were isolated from *C. rotang* leaf, was investigated against certain cancer and normal cells using the MTT assay. The cellular apoptotic activity and Panc1 cell cycle impact of compound (1) were examined through flow cytometry analysis and Annexin V-FITC cellular apoptotic assays. Additionally, RT-PCR was employed to evaluate the effect of compound (1) on the Panc1 apoptotic genes Casp3 and Bax, as well as the antiapoptotic gene Bcl-2. (Z)-3-hexenyl-*β*-_D_-glucopyranoside demonstrated the highest cytotoxic activity against Panc1 cancer cells, with an IC_50_ value of 7.6 µM. In comparison, gallic acid exhibited an IC_50_ value of 21.8 µM, and pyrogallol showed an IC_50_ value of 198.2 µM. However, (Z)-3-hexenyl-*β*-_D_-glucopyranoside displayed minimal or no significant cytotoxic activity against HepG2 and MCF7 cancer cells as well as WI-38 normal cells, with IC_50_ values of 45.8 µM, 108.7 µM, and 194. µM, respectively. (Z)-3-hexenyl-*β*-_D_-glucopyranoside (10 µM) was demonstrated to induce cellular apoptosis and cell growth arrest at the S phase of the cell cycle in Panc1 cells. These findings were supported by RT-PCR analysis, which revealed the upregulation of apoptotic genes (Casp3 and Bax) and the downregulation of the antiapoptotic gene Bcl-2. This study emphasizes the significant cellular potency of (Z)-3-hexenyl-*β*-_D_-glucopyranoside in specifically inducing cytotoxicity in Panc1 cells.

**Graphical Abstract:**

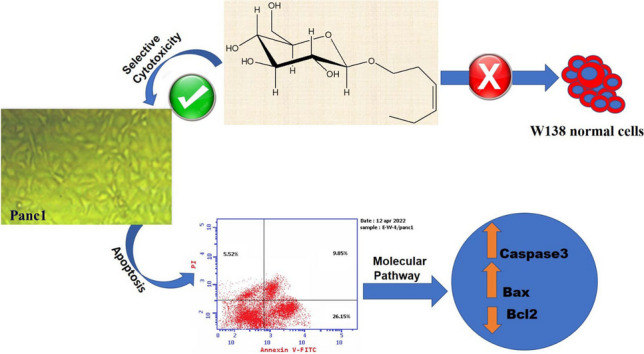

**Supplementary Information:**

The online version contains supplementary material available at 10.1007/s00210-023-02755-4.

## Introduction

Pancreatic cancer is one of the most fatal malignant tumors, posing a significant challenge in early diagnosis due to the absence of clear signs and symptoms. In advanced stages, pancreatic cancer exhibits a high mortality rate, with approximately 97% of patients expected to succumb within 5 years of diagnosis (Mario et al. 2018; Zhang et al. 2018). Disturbingly, the incidence, prevalence, and mortality rates of pancreatic cancer have all witnessed a global surge over the last 25 years, and projections indicate a further alarming increase of approximately 1.97-fold by 2060 (Mario et al. 2018).

Anticancer drugs continue to be a vital element in the treatment protocol for pancreatic cancer, whether used as standalone therapies or in conjunction with other modalities such as surgery, radiotherapy, and immunotherapy (Pliarchopoulou and Pectasides 2009; Neoptolemos et al. 2018).

Several plant extracts and metabolites have demonstrated remarkable cytotoxic activity against Panc1 cells, either when used alone or in combination with chemotherapeutic drugs. Notably, extracts derived from *Scutellaria barbata*, bitter apricot, *Moringa oleifera*, *Delonix regia*, *Mesua ferrea*, and the lipophilic fraction of *Senecio creuntus* have been reported to exhibit potent cytotoxic activity against Panc1 (Berkovich et al. 2013; Rajendran et al. 2016; Prescott et al. 2018; Wang et al. 2019; Aamazadeh et al. 2020; Awad et al. 2020; Shameli Rajiri et al. 2021; Malak et al. 2023).

Moreover, a variety of plant metabolites have exhibited potent cytotoxic activity against Panc1 cancer cells through diverse mechanisms. For example, berberine, a natural anticancer agent, has been identified as an inhibitor of Panc1 proliferation and an inducer of apoptosis (Rauf et al. 2021). Damnacanthal, isolated from *Garcinia huillensis*, has demonstrated the ability to induce necrotic death in Panc1 cells (Dibwe et al. 2012). Furthermore, curcumin, rhein, ellagic acid, embelin, metformin, and eruberin A are additional plant metabolites derived from various plant sources that exhibit cytotoxic effects against Panc1, with their respective mechanisms of action confirmed (Ramakrishnan et al. 2020). These findings suggest that further exploration of plant extracts and metabolites may lead to the discovery of novel sources of anticancer agents against Panc1, making them an area of significant scientific interest.

(Z)-3-hexenyl-*β*-_D_-glucopyranoside is a natural metabolite that has been extracted from various plants, including *Thymus vulgaris*, *Mallotus furetianus*, *Schizonepeta tenuifolia*, *Camellia sinensis*, *Xanthoxylum piperitum*, *Celosia argentea*, and *Epimedium glandiflorum* (Jiang et al. 2001; Kishida et al. 2005; Lee et al. 2008; Cui et al. 2016; Huang et al. 2018). Despite its prevalence in nature, there have been relatively few studies conducted on the biological activity of (Z)-3-hexenyl-*β*-_D_-glucopyranoside. One notable example is a study that identified antisteatotic activity in (Z)-3-hexenyl-*β*-_D_-glucopyranoside isolated from *Mallotus furetianus* (Huang et al. 2018).

Further research is warranted to delve into the potential biological activities and therapeutic applications of this natural metabolite.

## Experimental

### Solvents and chemicals for extraction and isolation of compounds

Hexane, methanol, dichloromethane, and ethyl acetate (Alfa chemicals, Cairo, Egypt) were employed for the extraction and isolation of metabolites. TLC plates (Merk, Darmstadt, Germany), RP-TLC plates (Merk, Darmstadt, Germany), normal column silica gel plates (Merk, Darmstadt, Germany), reversed phase C18 silica gel plates (Merk, Darmstadt, Germany), and Sephadex LH20 (Merk, Darmstadt, Germany) were utilized for the compound isolation process.

### Plant materials

**Fig. 1:**
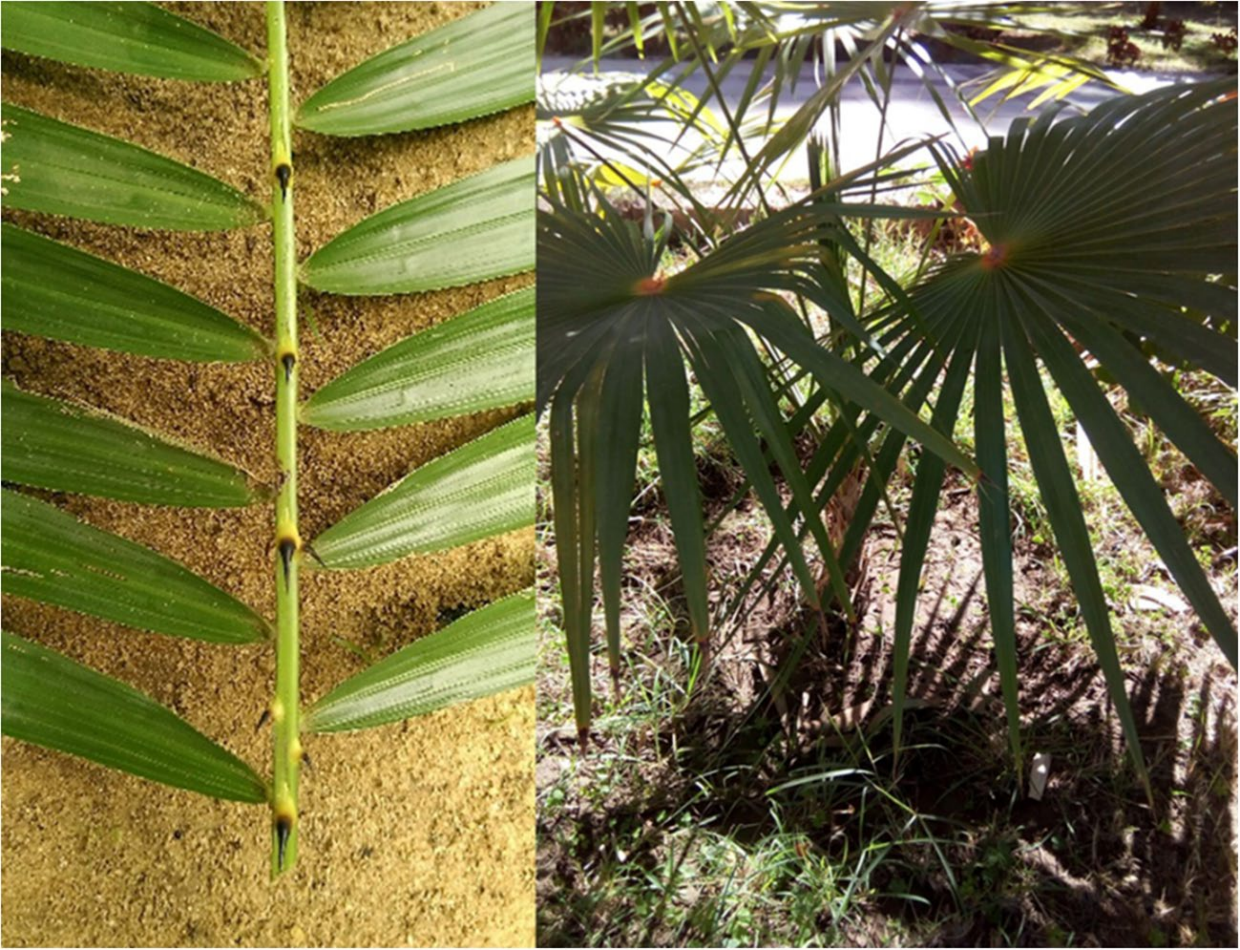
Photo of *C. rotang* leaf

The leaves of *Calamus rotang* L. (Fig. 1) were gathered from the Aswan Botanical Garden, located in Aswan, Egypt, in October 2018. Dr. Amr M. M. Mahmoud, the Director of Aswan Botanical Garden at the Horticultural Research Institute, Agriculture Center, Egypt, completed the taxonomical identification of the plant. A voucher specimen with the number A20220906 was deposited at the herbarium of the Department of Pharmacognosy, School of Pharmacy, Assiut University, Egypt.

### Isolation of compounds 1–3

The dried powdered leaves (2 kg) were subjected to maceration in 70% methanol (Alfa chemicals, Cairo, Egypt) (5 L × 3 times) at room temperature. The solvents were combined, and the resulting solution was concentrated under reduced pressure, yielding 300 g of residue. This residue was then suspended in 10% methanol (1 L) and fractionated using n-hexane (Alfa chemicals, Cairo, Egypt) (1 L × 6 times), dichloromethane (Alfa chemicals, Cairo, Egypt) (1 L × 6 times), and ethyl acetate (Alfa chemicals, Cairo, Egypt) (1 L × 6 times). Each solvent fraction was subsequently concentrated individually using a rotavapor (Buchi, Labortechnik AG, Flawil, Switzerland), resulting in the isolation of the n-hexane fraction (70 g), dichloromethane fraction (25 g), ethyl acetate fraction (32 g), and aqueous fraction (150 g). The dried ethyl acetate fraction was then subjected to chromatographic isolation of the bioactive compounds. In summary, the ethyl acetate fraction was subjected to sub-fractionation using chloroform: methanol solvent mixtures with varying percentages of methanol (0%, 5%, 10%, 15%, 20%, 30%, 50%, and 100%). Compound 1 (10 mg) was isolated from sub-fraction 4, which was eluted using 15% methanol in chloroform. Upon TLC analysis sprayed with 10% H_2_SO_4_, compound 1 exhibited a reddish-black spot. Purification of compound 1 was achieved through multiple open columns employing normal silica gel, RP-18, and Sephadex LH-20. Compounds 2 and 3 (100 mg and 200 mg, respectively) were isolated from sub-fraction 5, eluted using 20% methanol in chloroform. Both compounds 2 and 3 yielded brown spots on the TLC sprayed with 10% H_2_SO_4_ and were isolated using the same purification method as compound 1. The isolated compounds were subsequently dried and stored for NMR spectroscopic analyses (Bruker, Billerica, Massachusetts, USA) and biological assays.

### Cell culture

The cell lines PANC-1 (pancreatic carcinoma), MCF7 (breast carcinoma), HepG2 (hepatocellular carcinoma), and WI-38 (normal lung fibroblast cells) were obtained from the American Type Culture Collection (ATCC) located in Manassas, Virginia, United States. These cell lines were cultured in DMEM (Invitrogen, Life Technologies, Rockville, MD, USA) medium supplemented with 10% FBS (Fetal Bovine Serum, Hyclone, Thermo Fisher Scientific, Waltham, MA, USA) and 1% penicillin (Sigma-Aldrich, Louis, MO, USA). The cells were maintained at 37°C in a 5% CO_2_ and 95% humidity environment for a maximum period of two weeks to ensure their viability for further experimentation. Cell detachment was achieved using a solution containing 0.25% (w/v) trypsin (Sigma-Aldrich, Louis, MO, USA) and 0.53 mM EDTA ((Sigma-Aldrich, Louis, MO, USA), followed by re-cultivation in fresh media.

### MTT assay

The cultivated cells were seeded in 96-well plates at a concentration of 1 × 10^4^ cells/100 μL per well (Naik et al. 2014). The cultured plates were then incubated for 24 hours at 37°C. Stock solutions of the isolated compounds (1-3) and staurosporine (reference compound) were prepared at a concentration of 1 mg/mL in 10% DMSO (Sigma-Aldrich, Louis, MO, USA) in ddH_2_O. The tested concentrations of the compounds (1, 10, 30, and 100 µg/mL) in 0.01 - 0.1% DMSO in ddH_2_O were prepared by diluting the stock solutions with double-distilled water (ddH_2_O). After a 24-hour incubation of cells in 96-well plates, the media were replaced, and the tested concentrations of each compound, along with staurosporine, were added in triplicates (Esharkawy et al. 2022). A negative control using 0.1% DMSO in ddH_2_O was also included. The treated cells were further incubated for 48 hours at 37 °C. The media of the treated and control plates were removed and replaced by fresh media containing MTT (Sigma, Louis, MO, USA) reagent in a concentration of 1 mg/mL (Ponnusamy et al. 2016), then incubated for 2 hours. The viability of the cancer cells was determined by measuring the amount of formazan formed by viable cells. The produced formazan was solubilized by adding 100 µL 10% DMSO in ddH_2_O per each treated and control well. The plates were gently shacked for 5 min. The intensity of the produced colour was measured using an ELISA plate reader (Bio-Tek EL 800, Agilent technology, Santa Clara, CA, USA) at wavelength of 570 nm. The percent of cells viability was calculated by the following equation:

$$\mathrm{\%\; viability}={\mathrm{AA}}_{570}\mathrm{ \;of \;treated}-{\mathrm{AA}}_{570}\mathrm{ \;of \;blank}/ {\mathrm{AA}}_{570}\mathrm{ \;of \;control}-{\mathrm{AA}}_{570}\mathrm{ \;of \;blank}\times 100$$

AA: Average of triplicate absorbances for each sample concentration

Calibration curves of the tested and reference compounds were then prepared to calculate the IC_50_ (the concentration that inhibits 50% of cancer cells) (Esharkawy et al. 2022).

### Flow cytometry analysis of the cell DNA contents in Panc1 cells treated with (Z)-3-hexenyl-β-_D_-glucopyranoside

All reagents and kits for this assay were obtained from Abcam (Abcam Technology, Boston, MA, USA). The detection of DNA cell contents and cell cycle status was conducted following the established method (Xu et al. 2001). To provide a brief overview, Panc1 pancreatic cancer cells (ATCC, Manassas, Virginia, USA) were cultured in a single-cell suspension in DMEM medium (Invitrogen, Life Technologies, Rockville, MD, USA). Subsequently, the cells were fixed in 66% ethanol (Sigma-Aldrich, Louis, MO, USA) and kept on ice for 2 hours. Afterward, the cancer cells were washed with a PBS (Sigma-Aldrich, Louis, MO, USA) solution (5 mL of 10X PBS + 45 mL water), re-cultured in fresh medium, and incubated at 37 ºC for 24 hours. A concentration of 10 µM of (Z)-3-hexenyl-*β*-_D_-glucopyranoside was added in triplicate, chosen based on previously reported data (Ponnusamy et al. 2016; Xu et al. 2001) for 24h. The second dose of 10 µM of (Z)-3-hexenyl-*β*-_D_-glucopyranoside was added to the Panc1 cells then incubated for 24 h. Following a 48-hour incubation period from the first dose, the cells were trypsinized, fixed, and stained with propidium iodide-RNase enzyme reagent (9.45 mL PBS + 500 μL 20X propidium iodide + 50 μL 200X RNase) from Abcam Technology, Boston, MA, USA. The intensity of propidium iodide fluorescence, and thereby the amount of cellular DNA in each stage of the Panc1 cell cycle, was quantified using a flow cytometer (Novocyte, Agilent technology, Santa Clara, CA, USA) with an excitation maximum of 493nm and an emission maximum of 636 nm. The incubation time for fluorescence quantification was 30 minutes. The experiment was repeated twice.

### Annexin V-FITC cellular apoptotic assay

The reagents used in this assay included the Annexin V-FITC kit (Abcam Technology, Boston, MA, USA), 1X binding buffer (Abcam Technology, Boston, MA, USA), and propidium iodide (Bio Vision Research Products, Mountain View, CA, USA). Panc1 cells were seeded in 6-well plates and incubated at 37 ºC for 24 hours. Compound 1 ((Z)-3-hexenyl-*β*-_D_-glucopyranoside) was then added to the cultivated Panc1 6-well plates at a concentration of 10 µM and incubated for 48 hours. Subsequently, the cancer cells were trypsinized and centrifuged for 10 minutes at 300 rpm. The resulting precipitate was resuspended in 500 µL of 1X binding buffer. Annexin V-FITC (5 µL) and propidium iodide (5 µL) reagents were added to the treated cancer cell plates and incubated for 5 minutes in the dark at room temperature. A flow cytometer (Novocyte, Agilent technology, Santa Clara, CA, USA) with an excitation maximum of 488 nm and an emission maximum of 530 nm was employed to measure the intensity of annexin-binding phosphatidylserine (PS) and consequently determine the amount of apoptotic cells (Koopman et al. 1994).

### RNA isolation and SYBR® Green RT-PCR assay

The effects of 10 µM of (Z)-3-hexenyl-*β*-_D_-glucopyranoside on the mRNA expression of caspase3, bax, and Bcl-2 genes were investigated using the reverse transcription-polymerase RT-RNA technique. After a 48-hour incubation of (Z)-3-hexenyl-*β*-_D_-glucopyranoside in Panc1 (pancreatic carcinoma) cells, mRNA isolation of the selected proteins was performed using the RN easy extraction kit (Qiagen, Hilden, Germany), following previously described methods (Janicke et al. 1998). For real-time quantitative PCR of the RNA templates, a one-step RT-PCR kit with SYBR® Green (Bio-Rad SYBR Green PCR MMX, Hercules, California, USA) was added to a 25 μL 2X SYBR® Green RT-PCR reaction mix (Bio-Rad, Hercules, California, USA). The reaction mix contained 2X reaction buffer with 0.4 mM of each dNTP, magnesium chloride, iTaq DNA polymerase, 20 nM fluorescein SYBR® Green I dye, stabilizers, 11 μL nuclease-free water, 1.5 μL reverse primer (10 μM), 1.5 μL forward primer (10 μM), 1 μL iScript reverse transcriptase, and RNA template (1 pg to 100 ng total RNA). The cDNA synthesis and PCR amplifications were performed in the same tube using Rotor-Gene RT-PCR (Qiagen, Hilden, Germany) software 1.7 (Abed et al. 2021). The primers used in the RT-PCR reaction were obtained from Corbett Life Science Division (Reg. No. QEC21313), Corbett Research, Mortlake, Australia. The primer sequences used in the RT-PCR reaction were as follows:

- Caspase3: F 5’-GGAAGCGAATCAATGGACTCTGG-3’,
- Caspase3: R 5'-GCATCGACATCTGTACCAGACC -3',
- Bcl-2: F 5’-ATGTGTGTGGAGACCGTCAA -3’,
- Bcl-2: F 5’-GCCGTACAGTTCCACAAAGG -3’,
- Bax: F 5’-TCAGGATGCGTCCACCAAGAAG-3’,
- Bax: R 5’-TGTGTCCACGGCGGCAATCATC-3’

The Rotor-Gene RT-PCR reaction protocol was as follows: cDNA synthesis for 10 minutes at 50 ºC, iScript reverse transcriptase inactivation for 5 minutes at 95 ºC, PCR cycling and detection (30 to 45 cycles) for 10 seconds at 95 ºC, then 30 seconds at 55°C to 60°C (data collection step), and melt curve analysis for 1 minute at 95°C, 1 minute at 55°C, then 10 seconds at 55°C (80 cycles, increasing each by 0.5°C each cycle).

### Statistical analysis

The mean standard error of three measurements was used to calculate the IC_50_ of the tested reference compounds. ANOVA was used for statistical comparisons. P-values less than 0.05 were considered significant in the differences of treated cell lines by compounds 1-3 and reference stourosporine compared with the solvent control (0.1% DMSO).

## Results

### Identification of compounds 1–3

**Fig. 2:**
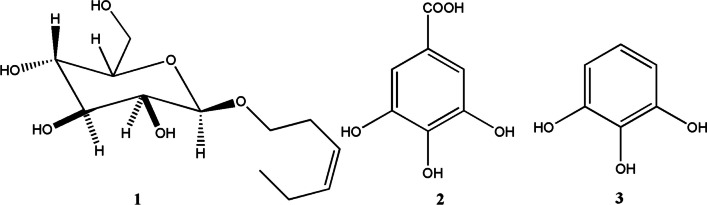
Chemical structure of compounds 1–3

The isolated compounds (Fig. 2) were identified by using NMR spectroscopy (Bruker 400 MHz, USA) and confirmed by a comparison with the previously published data.

- **(Z)-3-hexenyl-*β*-**_**D**_**-glucopyranoside (1)** (10 mg), colorless needle. ^1^H-NMR (Methanol-*d*_*4*_, 400 MHz) (Fig. S1): δ 5.50-5.36 (2H, m, H3, H4), δ 4.29 (1H, d, *J* = 7.76, H1'), 3.89-3.86 (2H, m, H6', H1), δ 3.70-3.66 (1H, m, H6'), δ 3.58-3.52 (1H, m, H1), δ 3.38-3.34 (3H, m, H2', H4', H5'), δ 3.21-3.16 (1H, dd, *J* = 7.96, 8.8 H3'), δ 2.40 (2H, dd, *J* = 6.96, 7.24, H2), δ 2.10 (2H, dq, *J* = 7.44, 7.36, H5), δ 0.98 (3H, t, *J* = 7.56). ^13^C-NMR (Methanol-d_4_, 100 MHz) (Fig. S2): δ 134.50 (C4), δ 125.85 (C3), δ 104.34 (C1'), δ 78.12 (C4'), δ 77.88 (C5'), δ 75.11 (C3'), δ 71.64 (C2'), δ 70.46 (C1), δ 62.75 (C6'), δ 28.78 (C2), δ 21.65 (C5), δ 14.62 (C6). The identification of compound 1 was confirmed by 2 D NMR (HSQC & HMBC) as shown in Figs. S3 and S4, in addition to a comparison the ^1^H and ^13^C-NMR data with those previous reported (Kishida et al. 2005).
- **Gallic acid (2)** (100 mg), white powder. ^1^H-NMR (DMSO-*d*_*6*_, 400 MHz) (Fig. S5): δ H 6.93 (2H, s, H2, H6). ^13^C-NMR (DMSO-*d*_*6*_, 400 MHz) (Fig. S6): δ 167.9 (C7), 138.51 (C4), 145.89 (C3, C5), 109.13 (C2, C6), 120.87 (C1). The compound was confirmed by a comparison the NMR data with those preciously reported (Nawwar et al. 1982).
- **Pyrogallol (3)** (200 mg), white amorphous powder.^1^H-NMR (400 MHz, DMSO-*d*_*6*_) (Fig. S7): δ 6.42 (1H, dd, *J* = 7.6, 7.6 Hz, H4), δ 6.27 (2H, d, *J* = 8 Hz, H5, H6). ^13^CNMR (100 MHz, DMSO-* d*_*6*_) (Fig. S8): δ 146.44 (C2, C6), 133.21 (C1), 118.72 (C4), 107.33 (C3, C5) (Liu et al. 1999).

### Cytotoxic activity of compounds 1–3

**Fig. 3:**
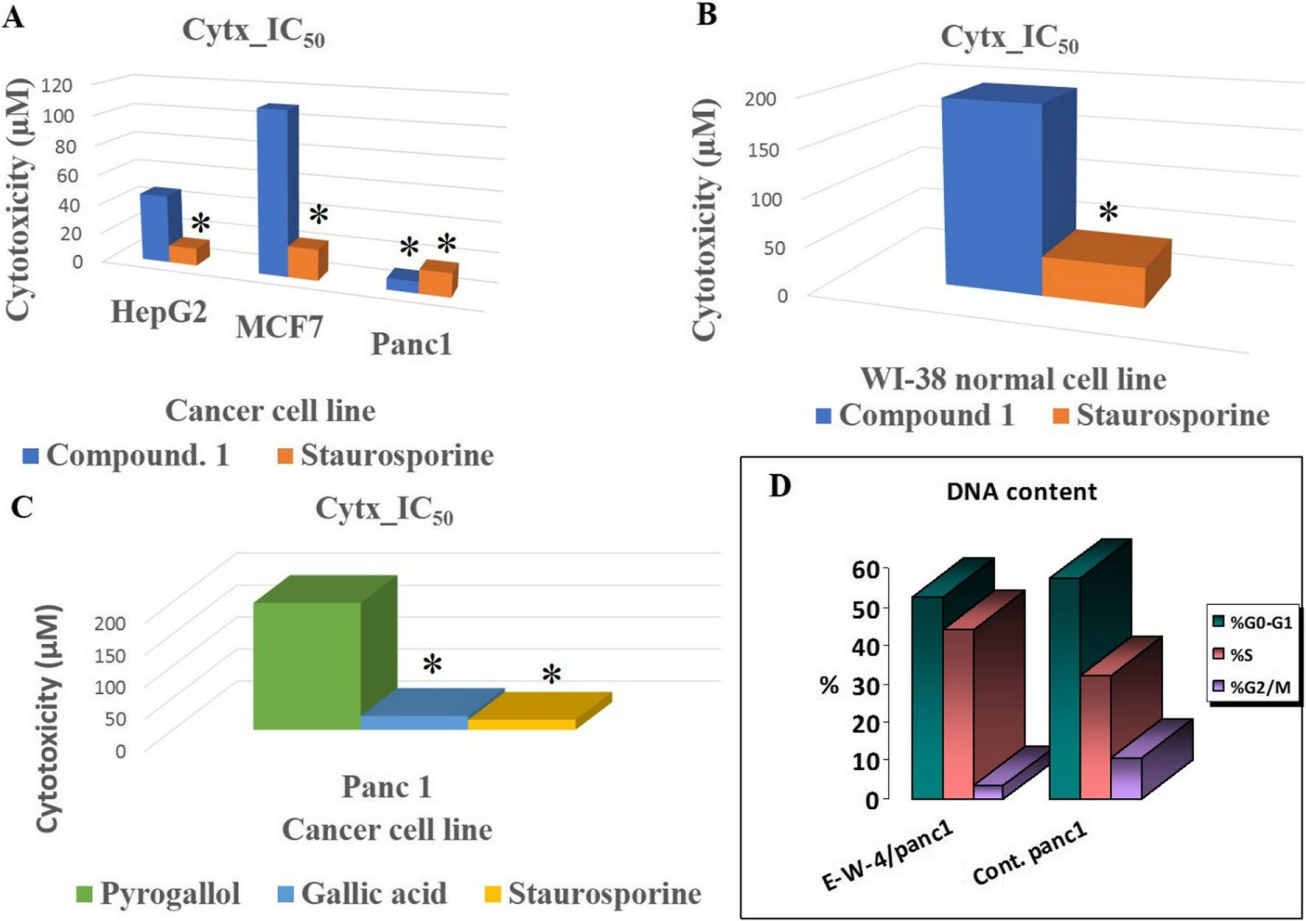
**A** Cytotoxic activity of (Z)-3-hexenyl-*β*-D-glucopyranoside (E-W-4) against HepG2, MCF7 and Panc1 cancer cells. **B** Cytotoxic activity of (Z)-3-hexenyl-*β*-D-glucopyranoside (E-W-4) against W138. **C** Cytotoxic activity of gallic acid (G) and pyrogallol (P) against Panc1 cancer cells. **D** Cell cycle profiling of treated panc1 cells with (Z)-3-hexenyl-*β*-D-glucopyranoside (E-W-4) and control panc1 cells. Statistically significant (P < 0.05) differences in treated cell lines by compounds 1–3 and reference stourosporine compared with the solvent control (0.1% DMSO) were indicated by asterisk symbol (*)

**Fig. 4:**
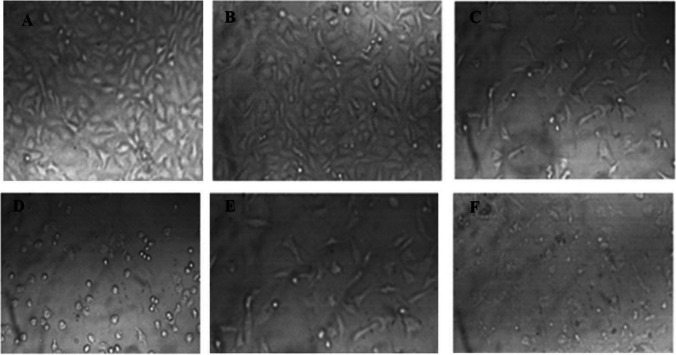
Representative images (40X) of Panc1 cells treated by different concentrations of (Z)-3-hexenyl-*β*-_D_-glucopyranoside compared to control (0.1% DMSO). **A**: Control Panc1 cells, **B**: 1 µg/mL treated Panc1 cells, **C**: 3 µg/mL treated Panc1 cells, **D**: 10 µg/mL treated Panc1 cells, **E**: 30 µg/mL treated Panc1 cells, **F**: 100 µg/mL treated Panc1 cells

The cytotoxic activities of the isolated compounds 1-3 are depicted in Figs. 3 and S2 and Table 1. In this study, we present the novel finding of the cytotoxic activity of (Z)-3-hexenyl-*β*-_D_-glucopyranoside (1) (Figs. 3A, B, and 4) against pancreatic (Panc1), hepatic (HepG2), and breast (MCF7) cancer cells, as well as WI-38 normal cells. It should be noted that compounds 2 and 3 have previously been reported as cytotoxic natural agents (Faried et al. 2007; Jiang et al. 2022; Maurya et al. 2010; Revathi et al.). In this study, we describe their cytotoxic activity against Panc1 cells, as shown in Table 2 and Fig. 3C.

**Table 1:** MTT cytotoxic activity (IC_50_) of (Z)-3-hexenyl-β-D-glucopyranoside (E-W-4) against HepG2 (Hepatic), MCF7 (Breast), Panc1 (Pancreatic) cancer cells and W138 normal cells

Ser	Cytotoxicity IC_50_ (µM)				
	Code	HepG2	MCF7	Panc1	WI38
1	Compound 1	45.8	108.7	7.6	194.6
***	Staurosporine	11.7	20.8	15.7	41

^***^ Reference compound, SD: Standard deviation ( ±)

**Table 2:** Cytotoxic activity (IC_50_) of gallic acid and pyrogallol against Panc1 cancer cells

No	Cytotoxicity IC_50_ (µM)	
	code	PANC1
1	Pyrogallol	198.2
2	Gallic acid	21.8
***	Staurosporine	15.7

^***^ Reference compound

### Panc1 cell cycle DNA arrest and apoptotic effects of (Z)-3-hexenyl-β-_D_-glucopyranoside

**Fig. 5:**
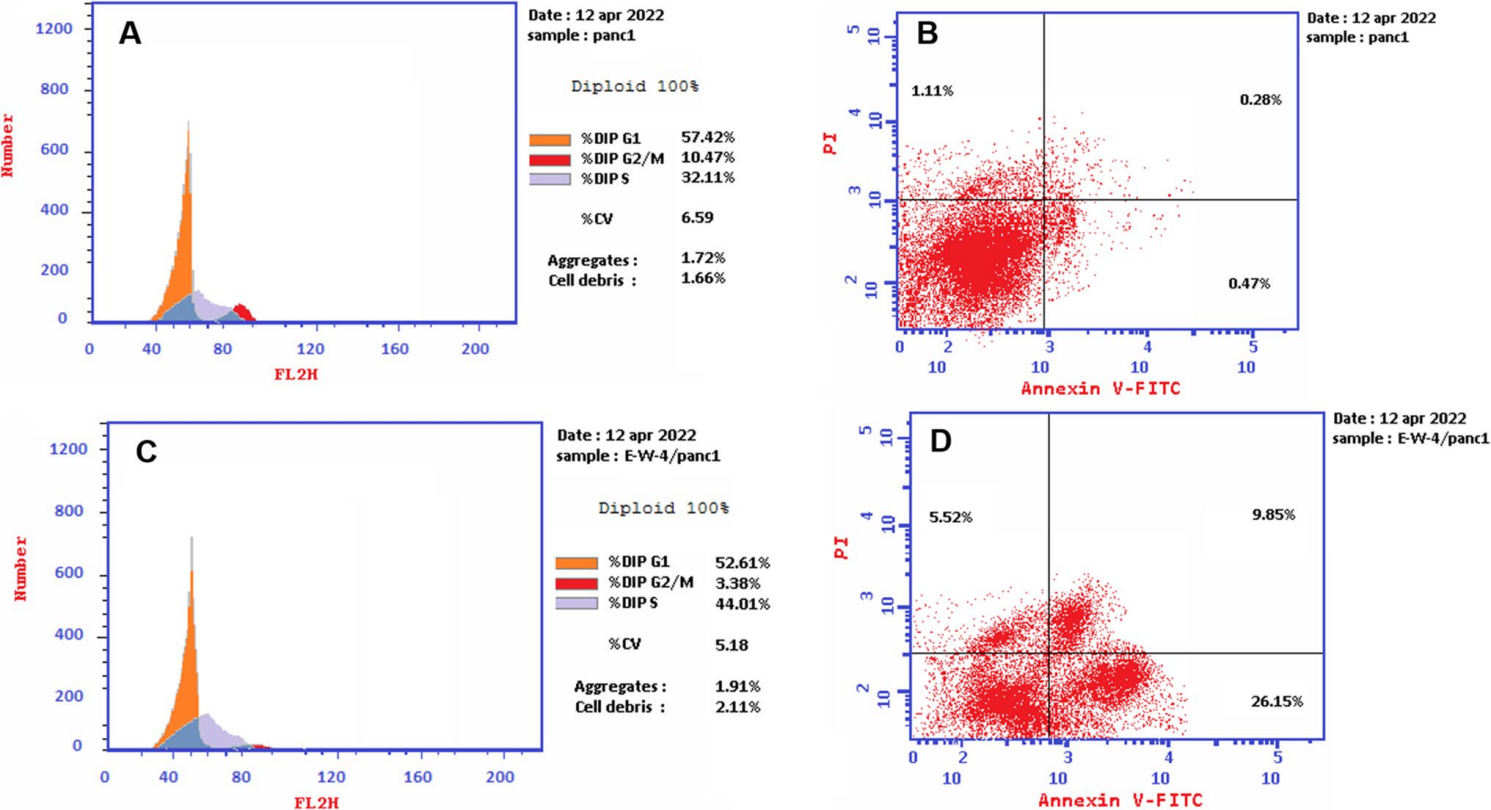
Apoptotic effects of (Z)-3-hexenyl-*β*-_D_-glucopyranoside (E-W-4) on Panc1 cells. **A** Cell count at various stages of controlled Panc1 cell cycle. **B** Annexin V/PI (Controlled Panc1). **C** Cell count at various stages of treated Panc1 cell cycle with 10 µM (Z)-3-hexenyl-*β*-_D_-glucopyranoside (E-W-4). **D** Annexin V/PI treated Panc1 with 10 µM (Z)-3-hexenyl-*β*-_D_-glucopyranoside (E-W-4)

Figures 3D and 5, along with Table S1, demonstrate that treatment of Panc1 cancer cells with (Z)-3-hexenyl-*β*-_D_-glucopyranoside (10 µM) resulted in cell growth arrest at the S phase and a significant decrease in cell DNA at the G2/M phase. Additionally, (Z)-3-hexenyl-*β*-_D_-glucopyranoside (10 µM) induced 26.15% early and 9.85% late apoptosis in Panc1 cells, as well as 5.52% necrosis (Table 3 and Fig. 5D).

**Table 3:** Cellular apoptotic effects of (Z)-3-hexenyl-*β*-_D_-glucopyranoside (10 µM) on Panc1 cancer cells

No	code	conc	Apoptosis		
			Total	Early	Late
1	Treated panc1		41.52%	26.15%	9.85%
2	Control panc1		1.86%	0.47%	0.28%

### Effect of (Z)-3-hexenyl-β-_D_-glucopyranoside on gene expression of caspase3, Bax and Bcl-2:

**Fig. 6:**
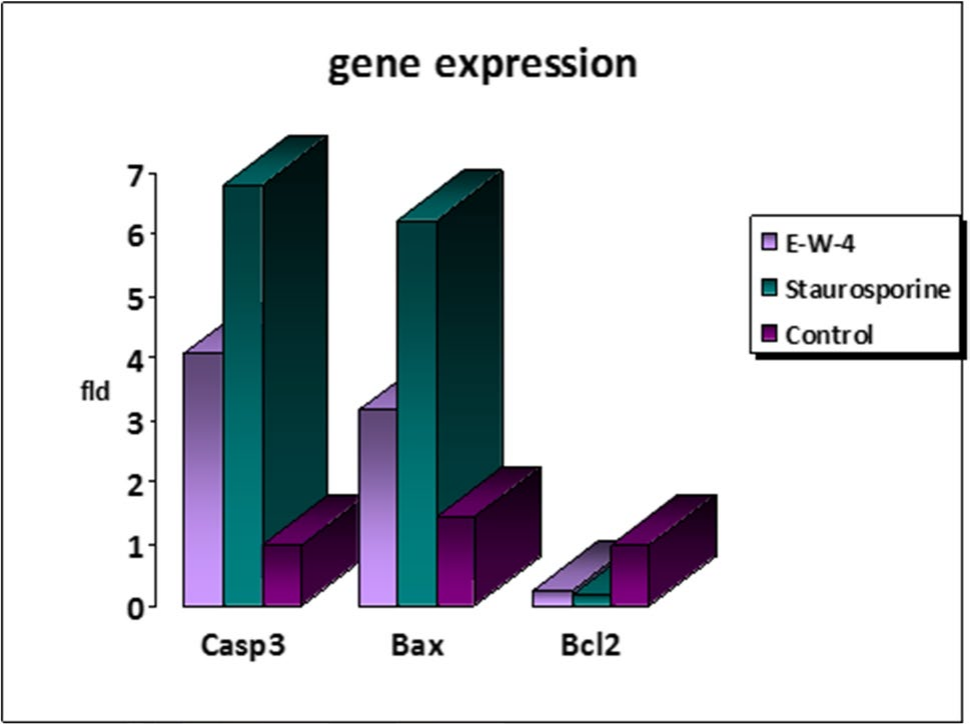
Caspase3, Bax and Bcl-2 gene expression levels in controlled Panc1 (untreated), positive controlled (Staurosporine treated) and tested Panc1 with 10 µM (Z)-3-hexenyl-β-_D_-glucopyranoside (E-W-4)

(Z)-3-hexenyl-*β*-_D_-glucopyranoside (10 µM) upregulated both caspase-3 and bax proapoptotic genes in Panc1 cells by 4.064 and 3.173-fold, respectively, while downregulating the Bcl-2 antiapoptotic gene by 0.231-fold (Table 4 and Fig. 6).

**Table 4:** Effects of (Z)-3-hexenyl-*β*-_D_-glucopyranoside (10 µM) on the caspase-3, bax and Bcl-2 gene expressions of Panc1 cancer cells

	Sample		RT-PCR results fold change		
No	code	Cells	Casp3	Bax	Bcl-2
1	(Z)-3-hexenyl-*β*-_D_-glucopyranoside	Panc1	4.064	3.173	0.231
2	Staurosporine	Panc1	6.794	6.217	0.183
3	Control	Panc1	1	1.436	1

## Discussion

The leaf ethyl acetate extract of *Calamus rotang* L. was subjected to isolation and characterization, resulting in the identification of compounds 1-3. In this study, we report the cytotoxicity of (Z)-3-hexenyl-*β*-_D_-glucopyranoside (1) for the first time, along with its molecular pathway. Additionally, two well-known natural anticancer compounds, gallic acid (2) and pyrogallol (3), were also isolated and characterized. (Faried et al. 2007; Tang et al. 2007; Maurya et al. 2010; Subramanian et al. 2015; Revathi et al. 2018, 2019; Jiang et al. 2022). These compounds have been previously reported and are commercially available, having been obtained from various medicinal plants (Khan et al. 2002; Verma et al. 2013). The compounds were identified based on NMR data in comparison with previously reported data (Nawwar et al. 1982; Liu et al. 1999; Kishida et al. 2005). (Z)-3-hexenyl-*β*-_D_-glucopyranoside (1) exhibited the highest cytotoxicity against Panc1 cells (IC_50_ = 7.6 µM) in a dose response manner. It was found to be twice as active as staurosporine (IC_50_ = 15.5 µM) and three times more active than gallic acid (IC_50_ = 21.8 µM) (Figs. 3 and 4, Table 1). However, it showed weak or no cytotoxicity against HepG2 and MCF7 cancer cells (IC_50_ = 45.8 and 108.7 µM, respectively) (Fig. 3A, Table 1). The cytotoxic activity of (Z)-3-hexenyl-*β*-_D_-glucopyranoside (1) on normal cells (WI-38) was also evaluated, and it exhibited weak to no cytotoxic activity (IC_50_ = 194.6 µM) compared to staurosporine (IC_50_ = 41 µM) (Fig. 3B, Table 1). Gallic acid (compound 2) demonstrated strong cytotoxic activity against Panc1 cells (IC_50_ = 21.8 µM), which is consistent with the previous reported data (Haddad and Rowland-Goldsmith 2014; Sharma et al. 2019). On the other hand, pyrogallol (compound 3) showed weak cytotoxicity against Panc1 cells (IC_50_ = 198.2 µM), although it has been reported to exhibit cytotoxic activity against other cancer cells (Mitsuhashi et al. 2008; Yang et al. 2009). Further investigation demonstrated that treatment with two doses of (Z)-3-hexenyl-*β*-_D_-glucopyranoside (10 µM) resulted in the induction of apoptosis in Panc1 cancer cells at the S1 stage of the cell cycle, as compared to the control Panc1 cells (Fig. 3D). This apoptotic effect was clearly observed in Fig. 5, where it is evident that (Z)-3-hexenyl-*β*-_D_-glucopyranoside caused a significant 42% increase in apoptosis in Panc1 cancer cells, with 26.15% early apoptosis and 9.85% late apoptosis. These findings are also summarized in Table 3.

Furthermore, the molecular analysis of the treated Panc1 cells with (Z)-3-hexenyl-*β*-_D_-glucopyranoside (10 µM) revealed notable changes in gene expression. Specifically, there was a significant increase in caspase-3 and Bax expression by 4.064 and 3.173-fold, respectively, indicating their involvement in the apoptotic pathway. Conversely, the expression of the antiapoptotic gene Bcl-2 decreased by 0.231-fold. These results are depicted in Figs. 3D, 5D, and 6, and further details can be found in Tables 3 and 4.

## Conclusion

The findings of this study strongly suggest that (Z)-3-hexenyl-*β*-_D_-glucopyranoside (1) holds great potential as an effective cytotoxic compound against Panc1 cells. Its mechanism of action involves inhibiting the proliferation of Panc1 cancer cells specifically during the S phase of the cell cycle through the induction of apoptosis. The observed upregulation of proapoptotic markers, such as caspase-3 and bax, along with the simultaneous downregulation of the antiapoptotic marker Bcl-2, provides further evidence of the cytotoxic activity of (Z)-3-hexenyl-*β*-_D_-glucopyranoside (1).

These findings shed light on the promising cellular activity exhibited by (Z)-3-hexenyl-*β*-_D_-glucopyranoside (1) in inducing cytotoxicity, particularly in Panc1 cells. Consequently, it is essential to conduct further investigations to explore its potential in vivo anticancer activity against pancreatic carcinoma. Future studies should focus on evaluating the efficacy and safety of (Z)-3-hexenyl-*β*-_D_-glucopyranoside (1) as a potential therapeutic agent for pancreatic cancer.

### Supplementary Information

Below is the link to the electronic supplementary material.

Supplementary file1 (DOCX 543 KB)

## Data Availability

Correspondence and requests for materials should be addressed to A.M.Z.

## Abbreviations

IC_50_: The half maximal inhibitory concentration
Bcl-2: B-cell lymphoma 2
Bax: Bcl-2-associated X protein
Casp3: Caspase 3
°C: Celsius temperature degree
µg/mL: Microgram/milliliter
Kg: Kilogram
Panc1: Pancreatic carcinoma
MCF7: Breast carcinoma
HepG2: Hepatocellular carcinoma
WI-38: Normal lung fibroblast cells
NMR: Nuclear Magnetic Resonance
^1^H-NMR: Proton-Nuclear Magnetic Resonance
^13^C-NMR: Carbon-Nuclear Magnetic Resonance
HMBC: Heteronuclear Multiple Bond Correlation
HSQC: Heteronuclear Single Quantum Coherence
PCR: Polymerase Chain Reaction
RT-PCR: Reverse Transcription Polymerase Chain Reaction
L: Liter
G: Gram
MHz: Megahertz
DMSO-*d*_*6*_: Deuterated dimethyl sulfoxide.
