# Supplementary material for: Potent anticancer activity of (Z)-3-hexenyl-β-D-glucopyranoside in pancreatic cancer cells
Source: Naunyn Schmiedebergs Arch Pharmacol. 2023 Oct 11;397(4):2311–20. doi: 10.1007/s00210-023-02755-4 (PMC10933169; doi:10.1007/s00210-023-02755-4)
Supplement: Supplementary file 1 — Supplementary file1 (DOCX 543 KB) [file 210_2023_2755_MOESM1_ESM.docx]

**SUPPLEMENTARY MATERIAL**

**Potent anticancer activity of (Z)-3-hexenyl-*β*-_D_-glucopyranoside in pancreatic cancer cells**

**Ahmed M. Zaher^1&3*^ Walaa S. Anwar^1^, Makboul A. Makboul^1^, Iman A. M. Abdel-Rahman^2^**

^1^ Department of Pharmacognosy, Faculty of Pharmacy, Assiut University, 71515

Assiut, Egypt.

^2^ Department of Pharmacognosy, Faculty of Pharmacy, South Valley University, Qena, Egypt.

^3^ Department of Pharmacognosy, Faculty of Pharmacy, Merit University, New Sohag, Egypt.

**Abstract**

**Purpose:** This current study reports, for the first time, on the potent cytotoxicity of (Z)-3-hexenyl-*β*-_D_-glucopyranoside, as well as its cellular and molecular apoptotic mechanisms against Panc1 cancer cells.

**Methods:** The cytotoxicity of three compounds, namely (Z)-3-hexenyl-*β*-_D_-glucopyranoside (1), gallic acid (2), and pyrogallol (3), which were isolated from *C. rotang* leaf, was investigated against certain cancer and normal cells using the MTT assay. The cellular apoptotic activity and Panc1 cell cycle impact of compound (1) were examined through flow cytometry analysis and Annexin V-FITC cellular apoptotic assays. Additionally, RT-PCR was employed to evaluate the effect of compound (1) on the Panc1 apoptotic genes Casp3 and Bax, as well as the antiapoptotic gene Bcl-2.

**Results:** (Z)-3-hexenyl-*β*-_D_-glucopyranoside demonstrated the highest cytotoxic activity against Panc1 cancer cells, with an IC_50_ value of 7.6 µM. In comparison, gallic acid exhibited an IC_50_ value of 21.8 µM, and pyrogallol showed an IC_50_ value of 198.2 µM. However, (Z)-3-hexenyl-*β*-_D_-glucopyranoside displayed minimal or no significant cytotoxic activity against HepG2 and MCF7 cancer cells as well as WI-38 normal cells, with IC_50_ values of 45.8 µM, 108.7 µM, and 194. µM, respectively. (Z)-3-hexenyl-*β*-_D_-glucopyranoside (10 µM) was demonstrated to induce cellular apoptosis and cell growth arrest at the S phase of the cell cycle in Panc1 cells. These findings were supported by RT-PCR analysis, which revealed the upregulation of apoptotic genes (Casp3 and Bax) and the downregulation of the antiapoptotic gene Bcl-2.

**Conclusion**: This study emphasizes the significant cellular potency of (Z)-3-hexenyl-*β*-_D_-glucopyranoside in specifically inducing cytotoxicity in Panc1 cells.

***** **Correspondence to**, Ahmed M. Zaher, Department of Pharmacognosy, Faculty of

Pharmacy, Assiut University, 71515 Assiut, Egypt, Email; [ahmedshaekhon@aun.edu.eg](mailto:ahmedshaekhon@aun.edu.eg) Tel.: +2-01023866348 (A.M.Z)

**Contents:**

**Fig S1:** ^1^HNMR spectral analysis of (Z)-3-hexenyl-β-D-glucopyranoside (1) (Methanol-d4, 400 MHz)

**Fig S2.:** ^13^CNMR spectral analysis of (Z)-3-hexenyl-β-D-glucopyranoside (1) (Methanol-d4, 100 MHz).

**Fig. S3:** HSQC spectral analysis of (Z)-3-hexenyl-β-D-glucopyranoside (1) (Methanol-d4, 400 – 100 MHz)

**Fig. S4:** HMBC spectral analysis of (Z)-3-hexenyl-β-D-glucopyranoside (1) (Methanol-d4, 400 – 100 MHz)

**Fig S5:** ^1^HNMR spectral analysis of gallic acid (2) (DMSO-*d6*, 100 MHz).

**Fig S6:** ^13^CNMR spectral analysis of gallic acid (2) (DMSO-*d6*, 100 MHz).

**Fig S7:** ^1^HNMR spectral analysis of pyrogallol (3) (DMSO-*d6*, 400 MHz).

**Fig S8:** ^13^CNMR spectral analysis of pyrogallol (3) (DMSO-*d6*, 100 MHz).

**Table S1:** Cell cycle examination of Panc1 cancer cells after 24h treatment with (Z)-3-hexenyl-β-D-glucopyranoside (E-W-4).

**
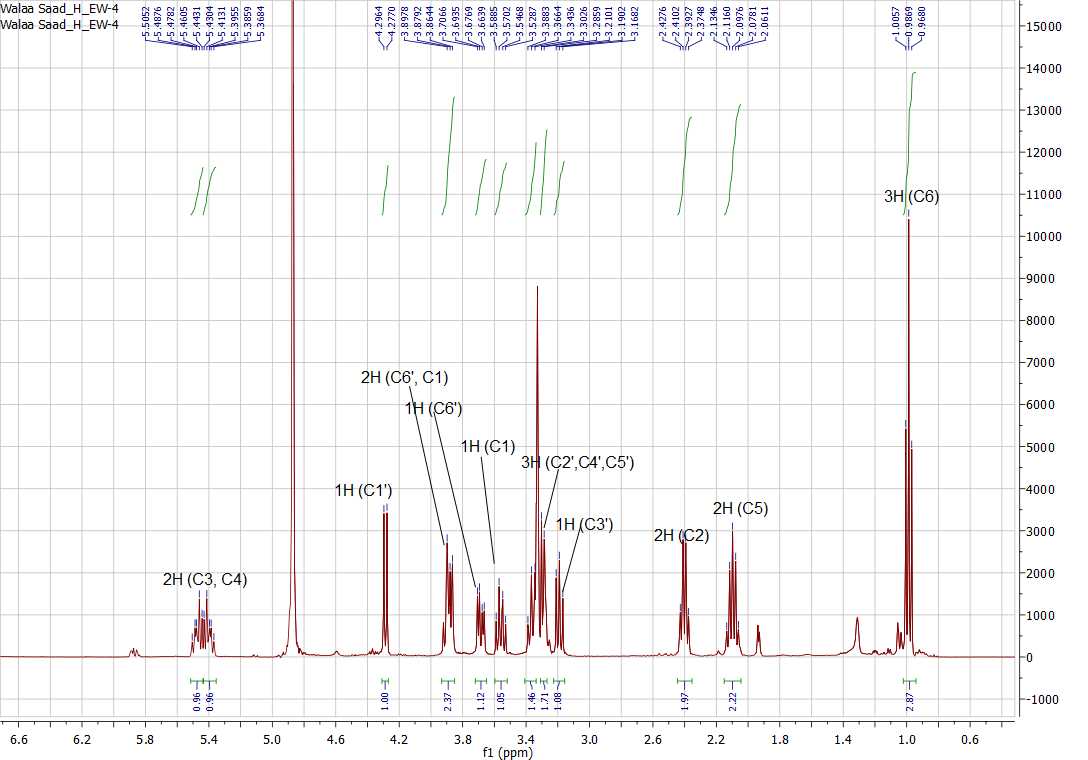
**

**Fig S1:** ^1^HNMR spectral analysis of (Z)-3-hexenyl-β-D-glucopyranoside (1) (Methanol-d4, 400 MHz)

**
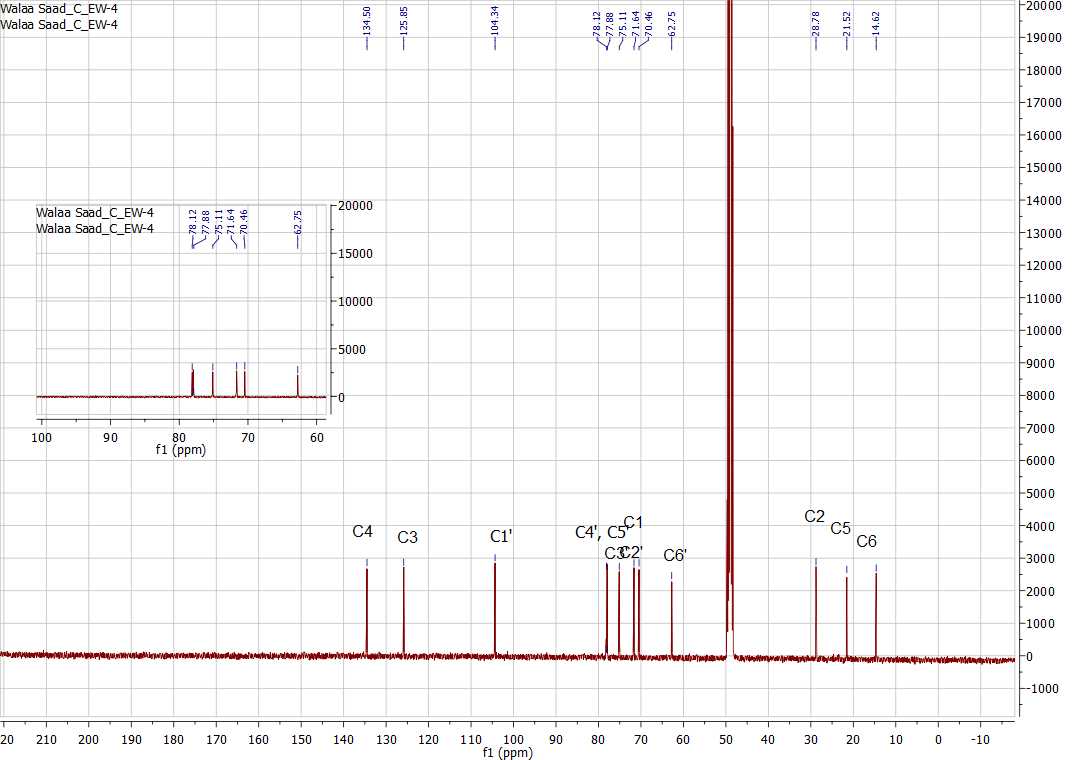
**

**Fig S2.:** ^13^CNMR spectral analysis of (Z)-3-hexenyl-β-D-glucopyranoside (1) (Methanol-d4, 100 MHz)

**
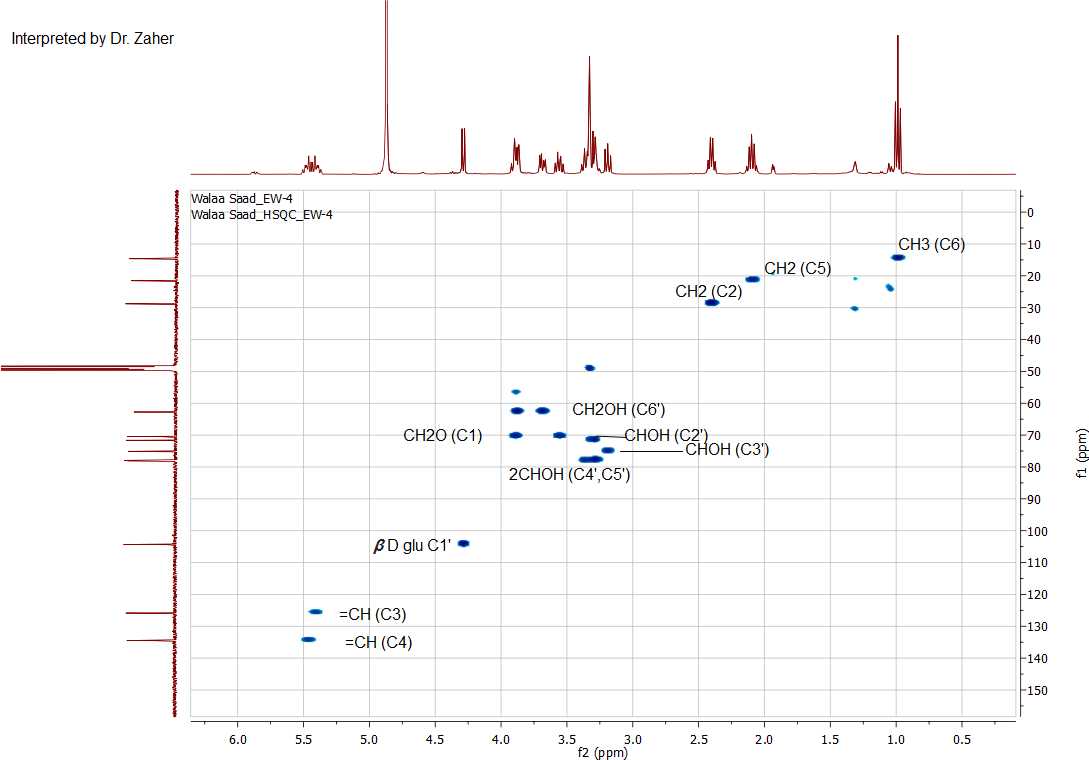
**

**Fig. S3:** HSQC spectral analysis of (Z)-3-hexenyl-β-D-glucopyranoside (1) (Methanol-d4, 400 – 100 MHz).

**
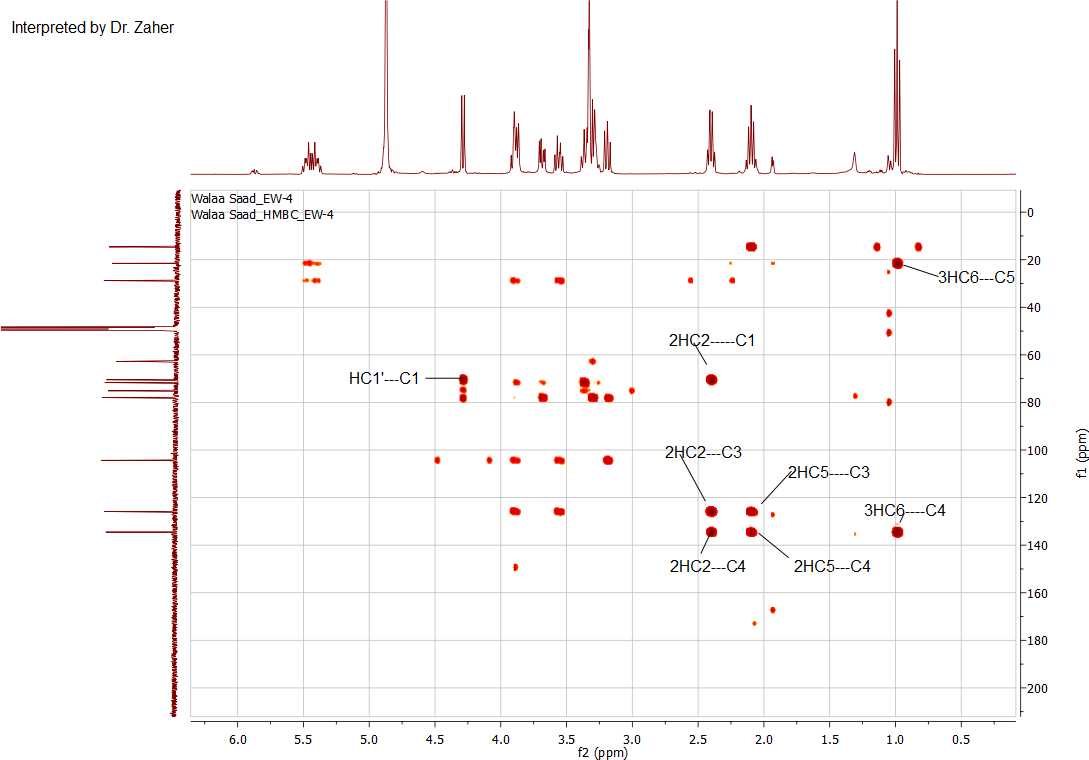
**

**Fig. S4:** HMBC spectral analysis of (Z)-3-hexenyl-β-D-glucopyranoside (1) (Methanol-d4, 400 – 100 MHz)


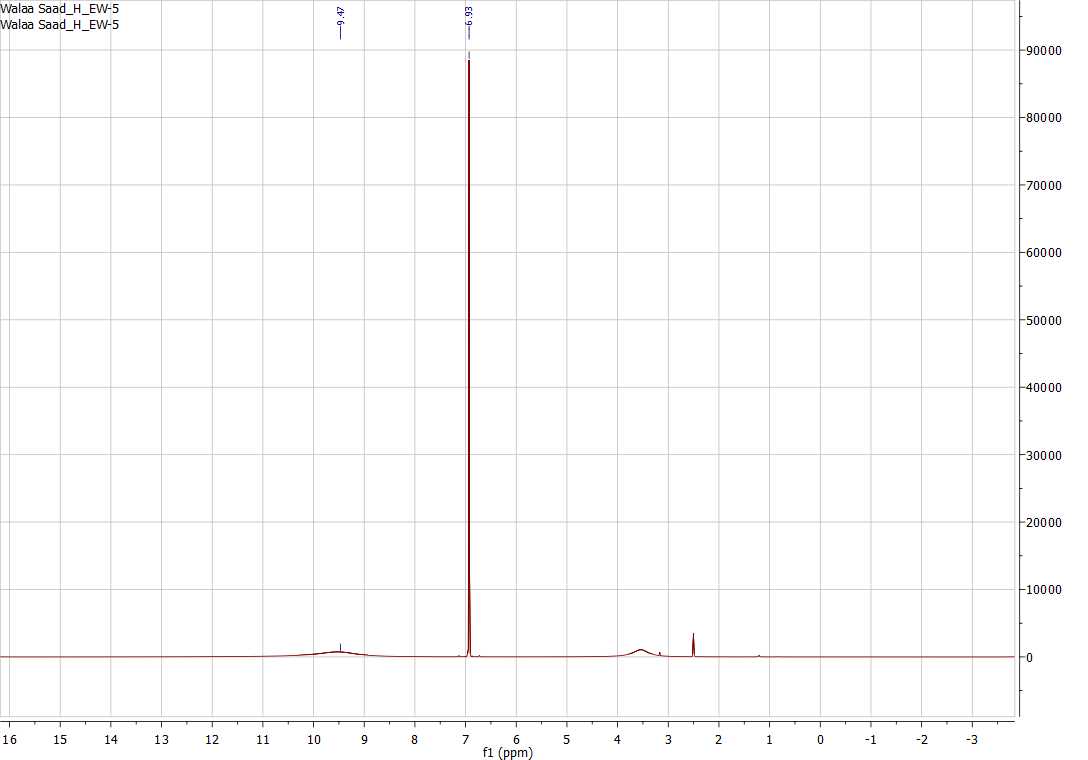


**Fig S5:** ^1^HNMR spectral analysis of gallic acid (2) (DMSO-*d6*, 100 MHz).


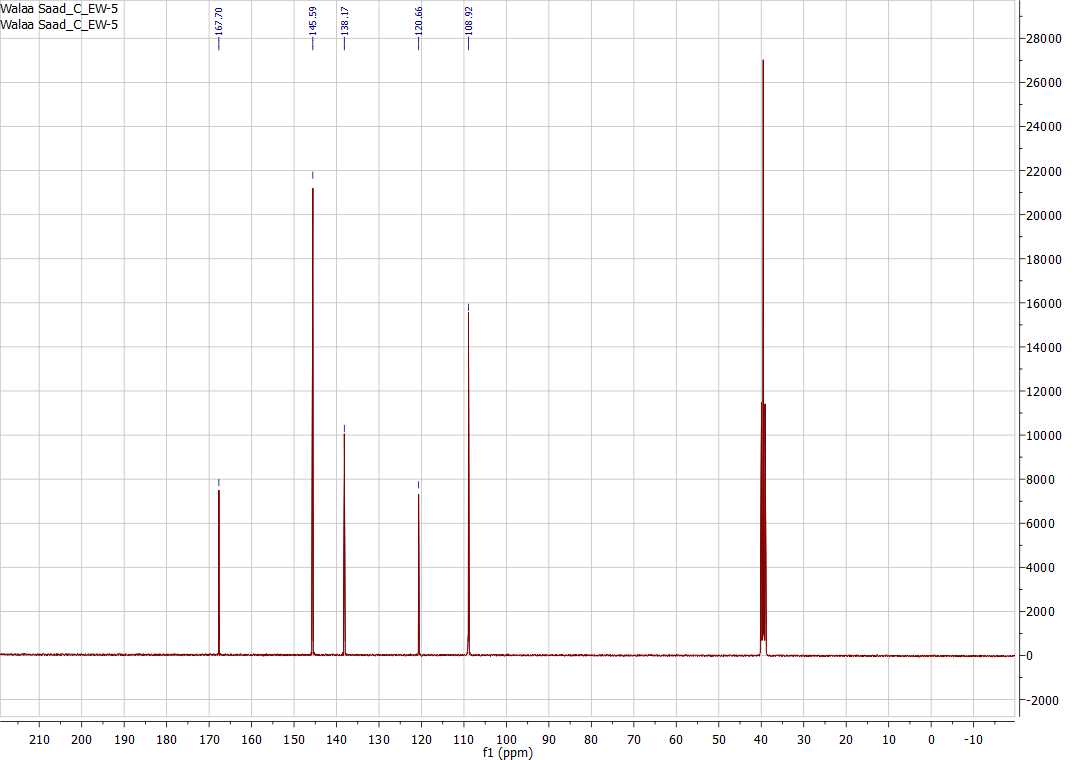


**Fig S6:** ^13^CNMR spectral analysis of gallic acid (2) (DMSO-*d6*, 100 MHz).


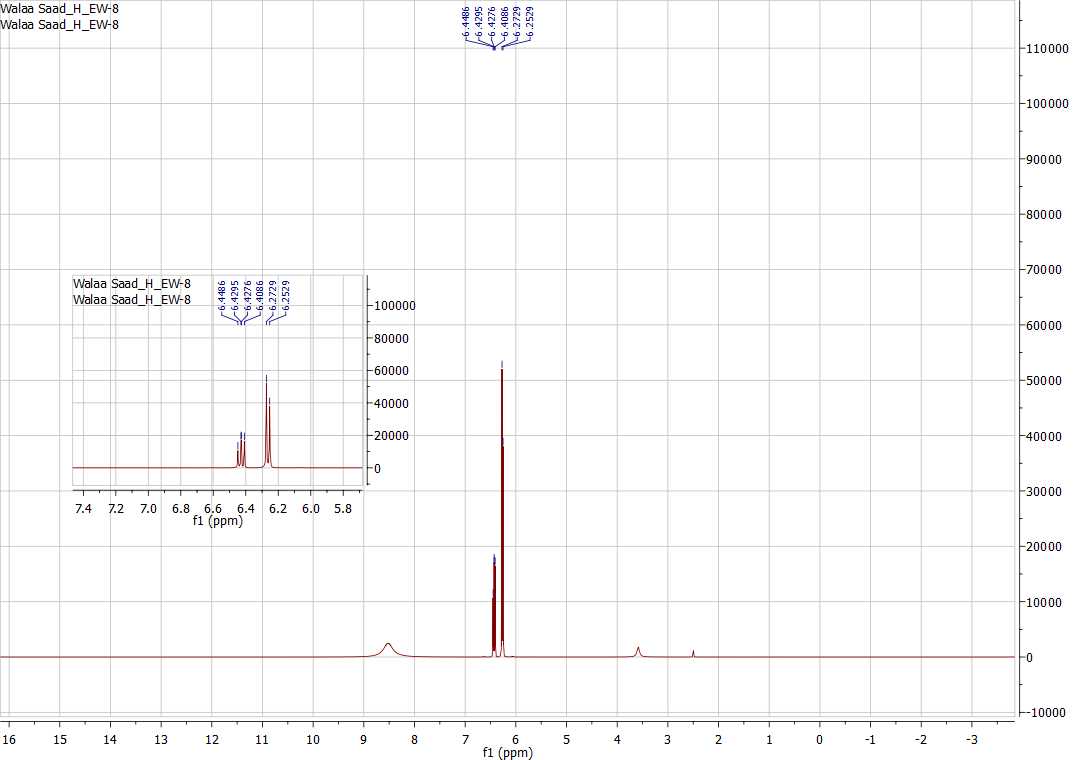


**Fig S7:** ^1^HNMR spectral analysis of pyrogallol (3) (DMSO-*d6*, 400 MHz).


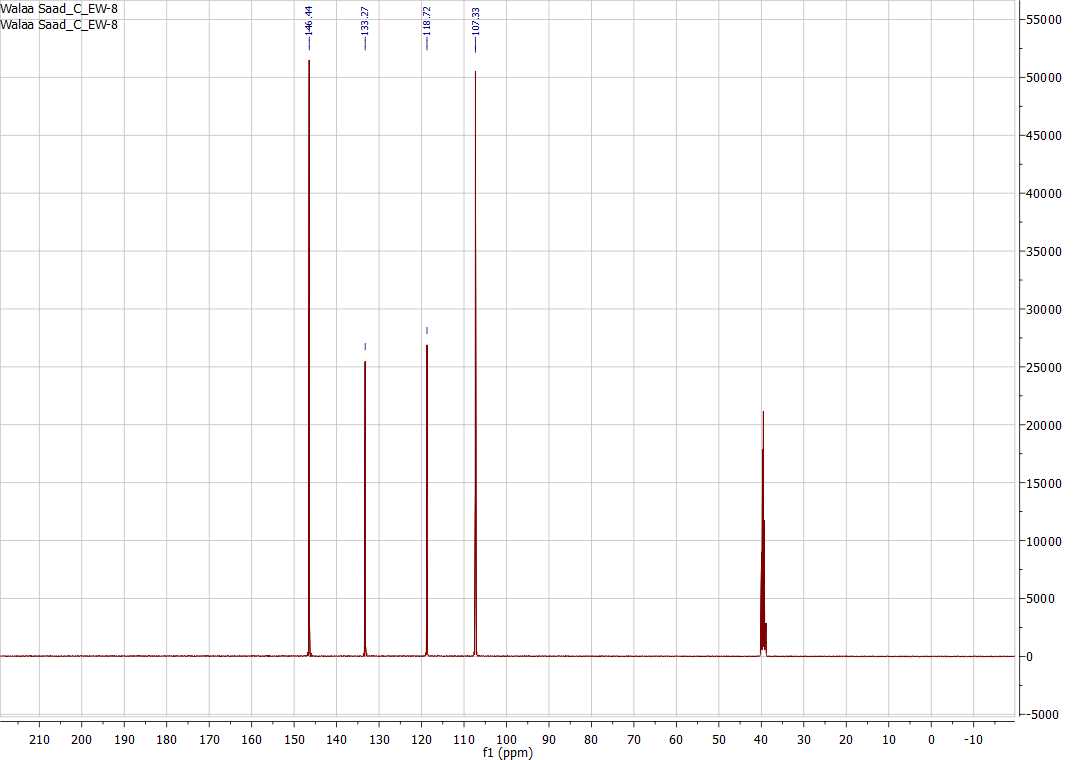


**Fig S8:** ^13^CNMR spectral analysis of pyrogallol (3) (DMSO-*d6*, 100 MHz).

**Table S1:** Cell cycle examination of Panc1 cancer cells after 24h treatment with (Z)-3-hexenyl-*β*-_D_-glucopyranoside (E-W-4).

| **No.** | **DNA content** | | | | |  |
| --- | --- | --- | --- | --- | --- | --- |
|  | **code** | **%G0-G1** | **%S** | **%G2/M** | **Comment** | |
| 1 | **E-W-4**/panc1 | 52.61 | 44.01 | 3.38 | Cell growth arrest@ S phase | |
| 2 | **Cont.** panc1 | 57.42 | 32.11 | 10.47 | --- | |
